# Supplementary figures and images for: Developmental Alcohol Exposure in Drosophila: Effects on Adult Phenotypes and Gene Expression in the Brain
Source: Front Psychiatry. 2021 Jul 22;12:699033. doi: 10.3389/fpsyt.2021.699033 (PMC8341641; doi:10.3389/fpsyt.2021.699033)

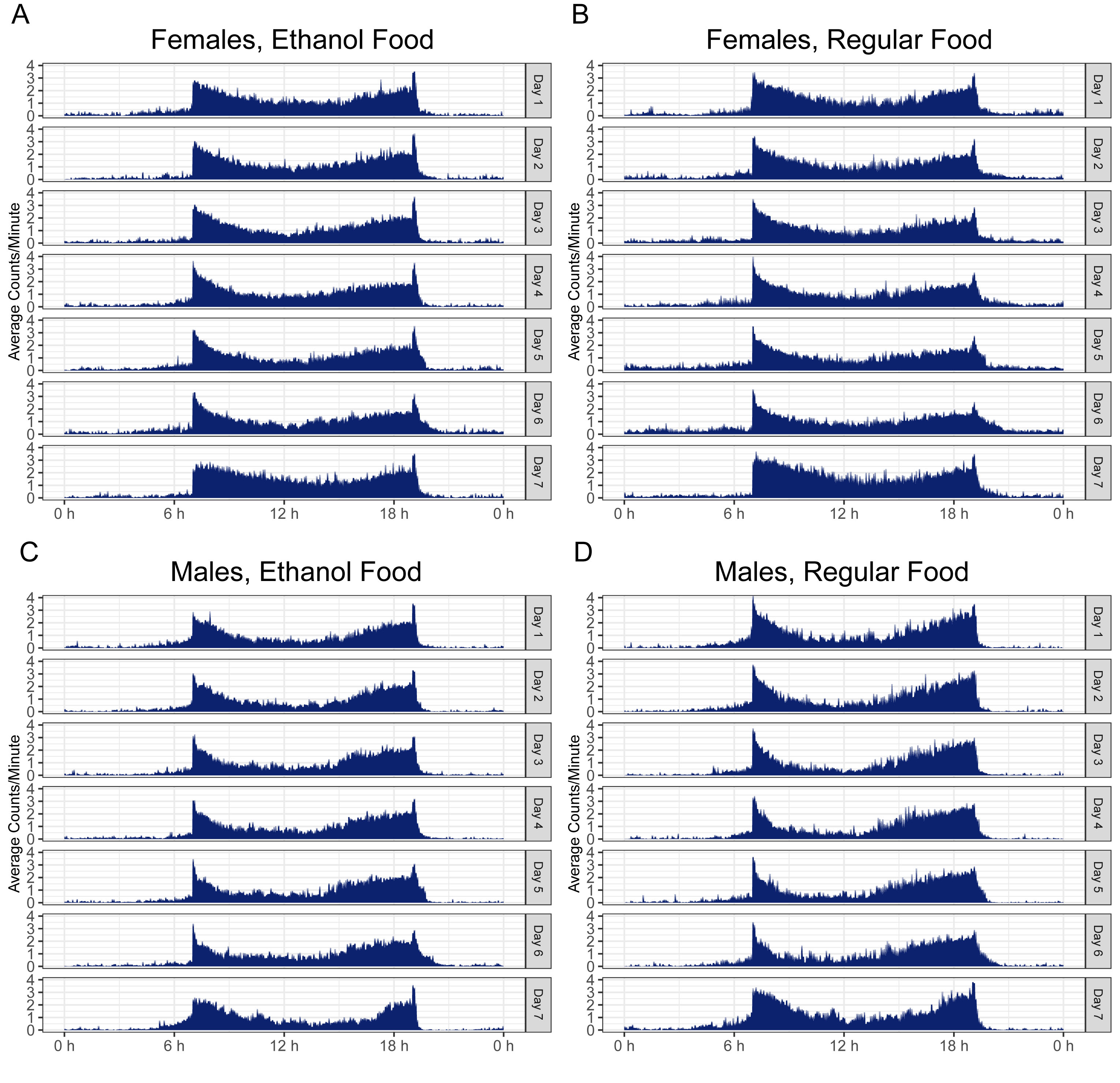

Supplement: Supplementary Figure 1 — Actograms showing average number of counts per fly per minute from females grown on (A) ethanol-supplemented food (10% v/v) and (B) regular food, and males grown on (C) ethanol-supplemented food (10% v/v) and (D) regular food. Actograms correspond to data collected for sleep and activity phenotypes shown in Figures 2D–G. Day hours are from 7:00 a.m. to 7:00 p.m., lights on 7 h after hour zero. Bin length = 5 min. [file Image_1.JPEG]

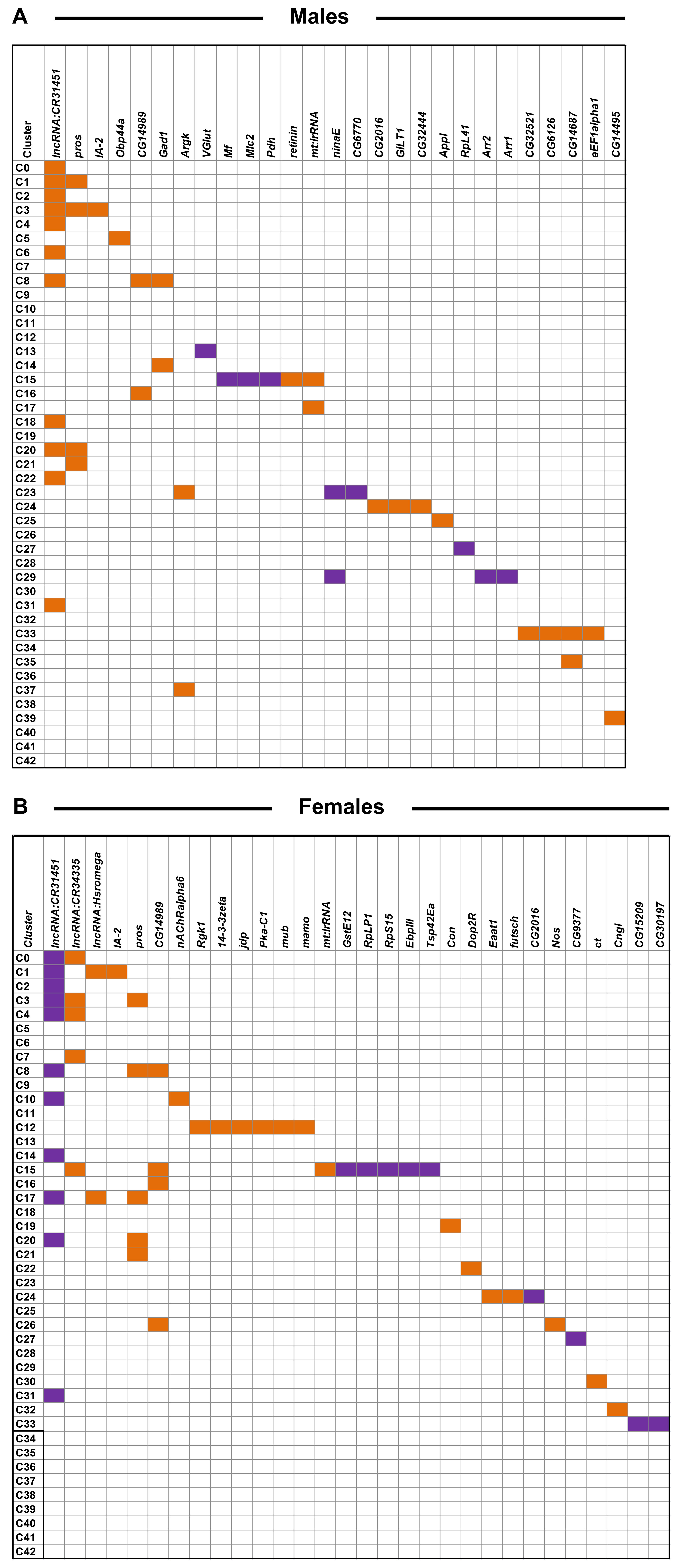

Supplement: Supplementary Figure 2 — Differentially expressed genes across clusters in males (A) and females (B) after developmental alcohol exposure. Differentially expressed genes are listed on the top (columns) and cell clusters are represented by the rows. Upregulated genes are indicated with orange and downregulated genes are indicated with purple. Differentially expressed genes are filtered at |logeFC| > 1.0 and a Bonferroni adjusted p < 0.05. [file Image_2.JPEG]
